# P-1722. Short versus Long Duration of Empiric Antifungal Use Among Patients with Sepsis or Septic Shock

**DOI:** 10.1093/ofid/ofae631.1886

**Published:** 2025-01-29

**Authors:** Thomas J Rust, Shawn Binkley, Michael DiCesare, Shannon Lawson, Vasilios Athans

**Affiliations:** Cleveland Clinic, Cleveland, Ohio; Hospital of the University of Pennsylvania, Philadelphia, Pennsylvania; Hospital of the University of Pennsylvania, Philadelphia, Pennsylvania; Hospital of the University of Pennsylvania, Philadelphia, Pennsylvania; Hospital of the University of Pennsylvania, Philadelphia, Pennsylvania

## Abstract

**Background:**

Invasive candidiasis is associated with high mortality rates among critically ill patients. There are currently no data to guide duration of empiric antifungal therapy (EAT) when indicated in high-risk patients with sepsis. The aim of this study was to evaluate clinical outcomes associated with a short versus long course of EAT in patients with sepsis or septic shock.

**Figure:**
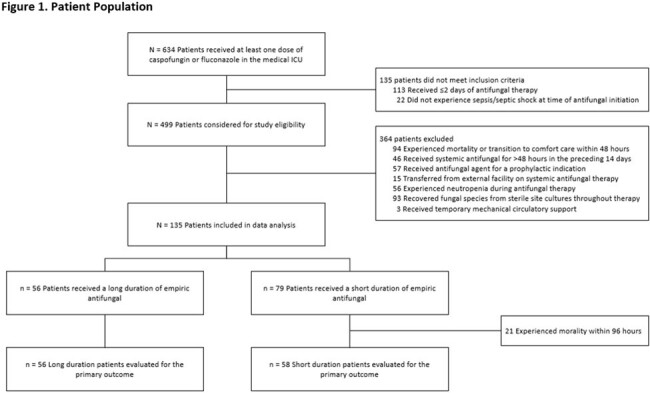

**Methods:**

This was a retrospective cohort study of adults admitted to the medical ICU who received > 2 days of EAT with fluconazole or caspofungin for sepsis or septic shock. Patients were excluded if they expired within 48 hours, had a positive fungal culture from a sterile site, were exposed to a systemic antifungal for > 48 hours in the 14 days preceding treatment, received prophylactic antifungals, or were neutropenic. In addition, patients who expired within 96 hours of EAT initiation were excluded from the primary outcome analysis. Patients were grouped according to receipt of EAT for ≤ 4 days (short duration) or > 4 days (long duration). The primary outcome was 30-day all-cause mortality. Secondary outcomes included time to mortality, hospital and ICU length of stay (LOS), vasopressor-free days over 14 days, and 30-day new proven fungal infection.

**Figure:**
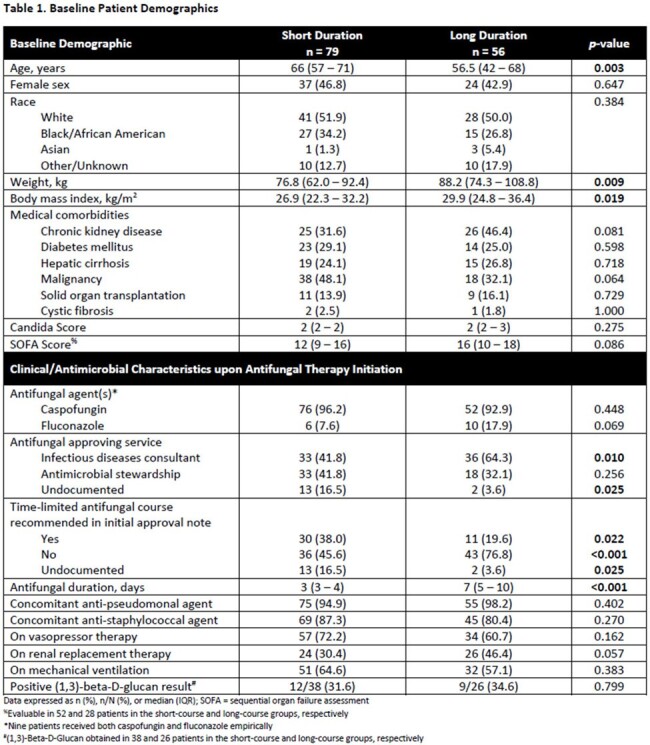

**Results:**

Of 135 included patients, 79 patients received short course EAT vs. 56 patients long course (Figure 1). Baseline variables were well balanced between groups apart from older age and lower body weight observed in the short duration group (Table 1). The primary outcome, evaluable in a total of 114 patients, was observed in 33 (56.9%) patients who received a short duration and 26 (46.4%) patients who received a long duration (*p*=0.263; OR 1.52, 95% CI [0.73 – 3.19]). There were no significant differences in all-cause inpatient mortality or time to mortality (Figure 2). Among patients who were discharged alive, there were no significant differences in secondary outcomes with the exception of fewer vasopressor-free days in the short course group (Table 2).

**Figure:**
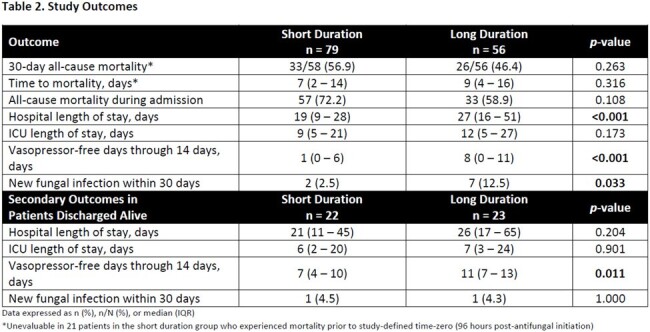

**Conclusion:**

Most clinical outcomes were not significantly different between patients with sepsis or septic shock who received a short versus long course of EAT. Duration of EAT is an important target for stewardship programs. This study adds to knowledge in the area, though a randomized trial may be necessary to substantiate our findings.

**Figure:**
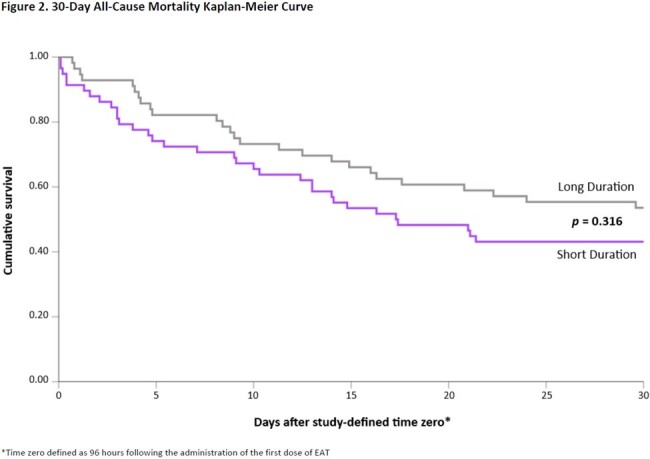

**Disclosures:**

**All Authors**: No reported disclosures

